# Metabotropic glutamate receptor 1 is associated with unfavorable prognosis in ER-negative and triple-negative breast cancer

**DOI:** 10.1038/s41598-020-79248-4

**Published:** 2020-12-18

**Authors:** Anna E. M. Bastiaansen, A. Mieke Timmermans, Marcel Smid, Carolien H. M. van Deurzen, Esther S. P. Hulsenboom, Wendy J. C. Prager-van der Smissen, Renée Foekens, Anita M. A. C. Trapman-Jansen, Peter A. E. Sillevis Smitt, Theo M. Luider, John W. M. Martens, Martijn M. vanDuijn

**Affiliations:** 1 Department of Neurology, Erasmus University Medical Center, Dr. Molewaterplein 40, 3015 GD Rotterdam, The Netherlands; 2 Department of Medical Oncology, Erasmus MC Cancer Institute, ′s-Gravendijkwal 230, 3015 CE Rotterdam, The Netherlands; 3 Department of Pathology, Erasmus MC Cancer Institute, ′s-Gravendijkwal 230, 3015 CE Rotterdam, The Netherlands; 4 Department of Neurology, Laboratory of Neuro-Oncology Clinical and Cancer Proteomics, Erasmus University Medical Center, Dr. Molewaterplein 40, 3015 GD Rotterdam, The Netherlands

**Keywords:** Prognostic markers, Cancer, Breast cancer

## Abstract

New therapies are an urgent medical need in all breast cancer subgroups. Metabotropic glutamate receptor 1 (mGluR1) is suggested as a potential new molecular target. We examined the prevalence mGluR1 expression in different clinically relevant breast cancer subgroups and determined its association with prognosis. In this retrospective cohort, 394 consecutive primary breast cancer tissues were incorporated into a tissue microarray and immunohistochemically stained for mGluR1. The prevalence of mGluR1 protein expression in different breast cancer subgroups was evaluated and correlated with metastasis-free survival (MFS) and overall survival (OS). In total, 56% (n = 219) breast cancer tissues had mGluR1 expression. In estrogen receptor (ER)-negative tumors, 31% (n = 18/58) had mGluR1 expression that was significantly associated with MFS (HR 5.00, 95% CI 1.03–24.35, *p* = 0.046) in multivariate analysis, independently from other prognostic factors. Of the 44 triple-negative breast cancer (TNBC), 25% (n = 11) expressed mGluR1. mGluR1 expression in TNBC was significantly associated with shorter MFS (HR 8.60, 95% CI 1.06–20.39, *p* = 0.044) and with poor OS (HR 16.07, 95% CI 1.16–223.10, *p* = 0.039). In conclusion, mGluR1 is frequently expressed in breast cancer. In ER-negative breast cancer and in TNBC mGluR1 protein expression is an unfavorable prognostic marker. This study provides rationale to explore mGluR1 as a novel target for breast cancer treatment, especially for the more aggressive TNBC.

## Introduction

Breast cancer is the most frequent and lethal cancer in women worldwide with approximately 2.1 million newly diagnosed breast cancer cases each year^1^. In the Western society, approximately one in eight women will develop invasive breast cancer during their life^2^. Although many women are effectively treated with specific therapies directed at the estrogen receptor (ER) and human epidermal growth factor type 2 receptor (HER2), 15% of breast cancers do not overexpress hormone receptors (ER and progesterone receptor (PR)) nor HER2 and are known as triple-negative breast cancer (TNBC). Women with TNBC cannot be treated with hormonal therapies nor therapies targeting HER2 and have a relatively poor outcome^3^.

ER and HER2 are not solely treatment targets in breast cancer. These receptors are also known prognostic markers as are age and tumor-related factors including tumor size, lymph node status and tumor grade^4–7^. A previous study identified tumor expression of metabotropic glutamate receptor 1 (mGluR1/GRM1) as another possible prognostic marker^8^. This study was the first to show that ER-positive breast cancer is significantly more likely to express mGluR1 than ER-negative tumors. In another dataset of primary ER-positive and adjuvant tamoxifen-treated patients, low GRM1 expression associated with a longer distant metastasis-free survival (MFS) as compared to higher GRM1 expression^8^. The prognostic value of mGluR1/GRM1 expression in other breast cancer subgroups is unknown.

Metabotropic glutamate receptors are a family of G-protein coupled receptors, that bind the excitatory neurotransmitter glutamate; mGluR1 belongs to the mGluR Group I family^9,10^. Although mGluR1 is best known for its role in nervous system development, function and pathology, by mediating neuronal excitability and synaptic plasticity, its significance in tumorigenesis has been demonstrated^11–14^. Overexpression of mGluR1 in a mouse model unexpectedly produced melanomas with 100% penetrance^11^. Subsequently, it was demonstrated that mGluR1 was expressed in more than 60% of human melanoma tissues^15^. Currently it is known that mGluR1 is expressed in substantial proportions of breast cancer, renal cell cancer and prostate cancer with indications that mGluR1 functions as an oncogene in these tumors^8,12,13,16,17^. In addition to being a prognostic marker, mGluR1 may also serve as a novel therapeutic target for the treatment of many cancers including breast cancer^13,18^. Despite the promise of mGluR1 as prognostic marker^8^ and potential treatment target in breast cancer^13,18^, all published studies are based on relatively small cohorts and have not been validated.

In the current study, we evaluated mGluR1 expression in a not previously described cohort consisting of consecutive primary breast cancer patients. Additionally, we aimed to determine the prognostic value of mGluR1 expression in hormone receptor and/or HER2 positive and negative breast cancer subgroups in this independent and representative cohort.

## Methods

### Patients, tumor tissue samples and databases

**Figure 1:**
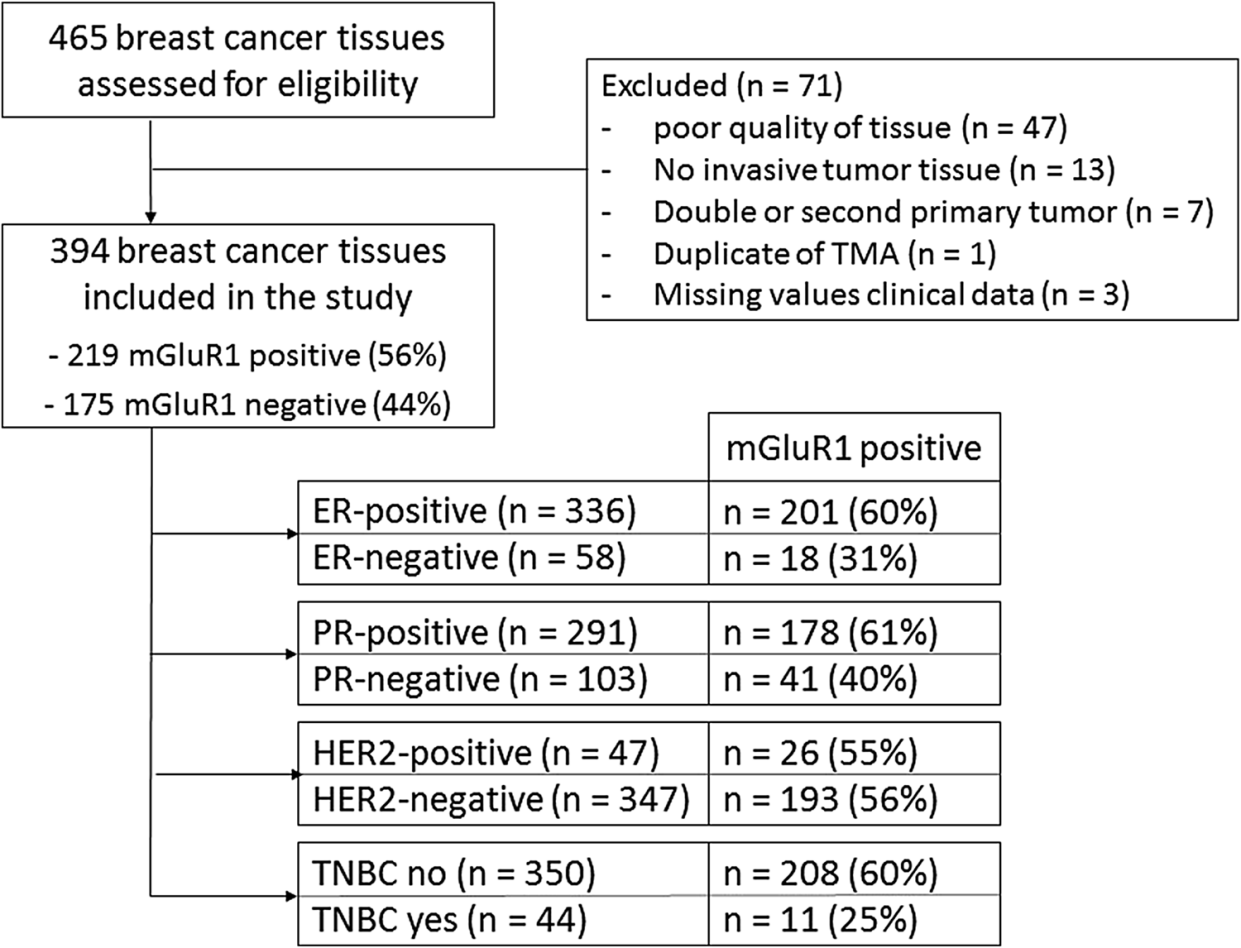
Flowchart of primary breast cancer tissues included in this study.

In this retrospective study, all tissues were derived from consecutive patients with primary breast cancer who were operated in the Erasmus University Medical Center from 2000 through 2005. In total 465 eligible tumors were analyzed. 71 cases were excluded due to missing values and other factors depicted in Fig. 1, resulting in a final number of 394 tissues for data analysis. Of all these patients complete clinical follow-up information was available until 2017. This study was performed in accordance with the Code of Conduct of the Federation of Medical Scientific Societies in the Netherlands (http://www.federa.org/codes-conduct) and used coded tumor tissues remaining after the routine procedure to diagnose the disease. According to this Code of Conduct, no informed consent is needed for this study. This procedure, including the need for informed consent, was waived and approved by the Medical Ethics Committee of the Erasmus MC (approval number MEC 02.953). Reporting Recommendations for Tumor Marker Prognostic Studies were followed (REMARK)^19^.

To confirm the prognostic value of mGluR1 at the RNA level, we consulted (partially) publicly available data from four independent breast cancer cohorts: (1) Affymetrix U133-A array data of 866 breast cancer patients, including 142 with TNBC^20^, (2) TCGA data from which 166 TNBC cases were extracted^21,22^, (3) RNAseq data of 41 TNBC from Erasmus MC cohort (Hammerl D. et al. unpublished data), (4) RNAseq data of 36 TNBC patients from BASIS cohort containing 266 primary breast cancer cases^23^.

### Tissue microarray and immunohistochemical evaluation

Formalin-fixed paraffin-embedded (FFPE) tissue blocks were selected to be captured in tissue microarray (TMA) by using hematoxylin/eosin-stained tissue sections. A breast pathologist graded the eligible tumors, determined the histological subtype, and marked three representative areas of the tumors. From each of the three selected areas, a core was extracted with a 0.6 mm needle and put in a blank receiver block (280 cores per block) using the Automated Tissue Arrayer ATA-27 (Beecher Instruments Inc, Sun Prairie, WI). Tumor grade was defined according to the modified Bloom-Richardson score^7^, and histology type was assessed on whole tissue sections prior to preparing the TMA^24^.

**Figure 2:**
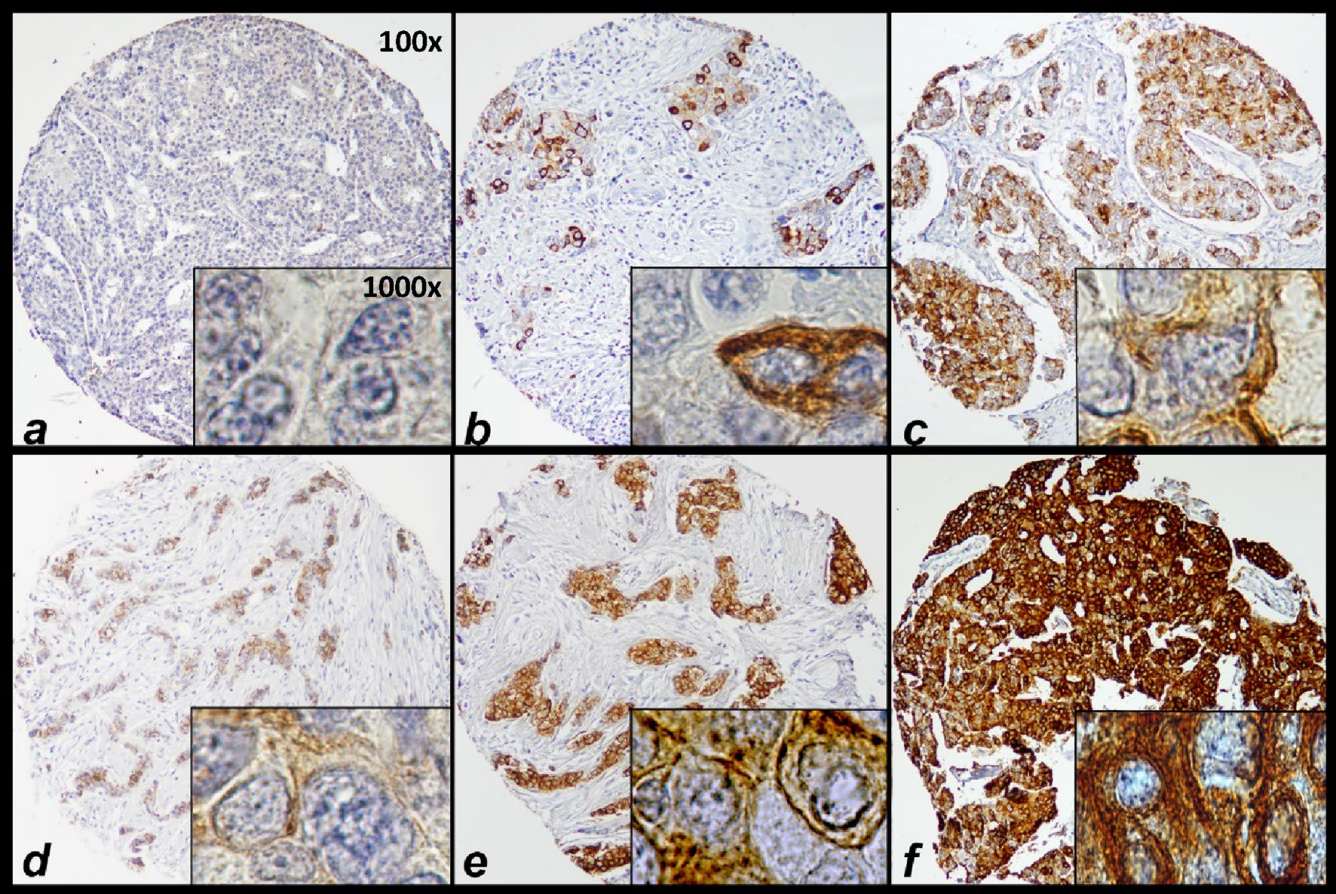
Scoring of immunohistochemical mGluR1 staining on TMA. Representative examples of mGluR1 staining in breast cancer tissues are shown. In these examples staining quantity (% of tumor cells) is scored as follows: (**a**) negative; (**b**) 30%; (**c**) 75%; and (**f**) 100%. Intensity is scored as follows: (**d)** weak; (**e**) moderate; and (**f**) strong.

Sections from each TMA block were cut (4 µm) and stained for ER, PR, mGluR1 and HER2 expression in triplicate. Primary antibodies and used conditions are listed in Additional file 1. ER, PR and mGluR1 antibodies were stained with the EnVision method (Dako, Glostrup, Denmark) and the HER2 staining with the HercepTest (Dako). Within the TMA, positive and negative control tissues were included containing tumor cell lines with known levels of HER2, ER and PR expression. mGluR1 transfected and untransfected Chinese hamster ovary (CHO) cells were used as controls for mGluR1 staining^25^. After staining, slides were converted to digital images using the Virtual Slide Scanner NanoZoomer 2.0-HT (Hamamatsu, Hamamatsu-City, Japan). These digital images were uploaded in our TMA database (Distiller, Slidepath, Dublin, Ireland). For mGluR1 the staining quantity (percentage of positive stained tumor cells) and intensity (0 = negative, 1 = weak, 2 = moderate, 3 = strong) was independently scored and consolidated by well trained technicians (Fig. 2). Only staining of invasive tumor cells was scored and scoring was done manually for all images. The percentage of the positively stained breast tumor cells was calculated relative to the total tumor cell count of 3 separate cores per tumor. mGluR1 was considered positive if > 25% of tumor cells had a membrane (diffuse and/or apical) staining pattern. Positive ER and PR cells were recorded only if staining was seen within the nuclei and the cut-off used to classify tumors as ER-positive or PR-positive was > 10% positive tumor cell nuclei. HER2 was considered positive for moderate to strong (3 +) intact membranous staining of > 30% of the tumor cells. HER2 staining tumors with a weak to moderate (2 +) staining aspect (and > 10% of the tumor cells) were additionally subjected to fluorescence in situ hybridization with the HER2FISH pharmDx Kit (Dako) and scored according to the kit guidelines. These tumors were considered HER2 positive when the HER2 gene was amplified.

### Statistical analysis

Statistical analysis was performed using IBM SPSS Statistics 25. The relationship of patient and tumor characteristics with mGluR1 expression in the different subgroups was investigated using the Pearson Chi-Square test or the Fisher-Exact test when appropriate. In the entire cohort and in relevant subgroups univariate and multivariate Cox regression analysis was performed to identify which factors were associated with survival and for the different variables their hazard ratio (HR) with 95% confidence interval (95% CI) were calculated. For survival, metastasis-free survival (MFS) and overall survival (OS) were used as end points with MFS defined as the time from diagnosis to the first distant metastasis and OS as the time from diagnosis until patient’s death. All traditional risk factors were included in the model and mGluR1 expression was added to this base model. The Kaplan–Meier method was used for generating survival curves to visualize MFS and OS. Log-rank tests were used to test for differences between survival curves. Kaplan–Meier estimates and Log-rank tests were also used for relationship analysis between intensity of mGluR1 staining or a percentage of positively stained tumor cells (high vs low in both instances) and survival (MFS and OS). All *p* values were two sided and were considered statistically significant when below 0.05.

### Ethics approval and consent to participate

This study was performed in accordance with the Code of Conduct of the Federation of Medical Scientific Societies in the Netherlands (http://www.federa.org/codes-conduct) and used coded tumor tissues remaining after the routine procedure to diagnose the disease. According to this Code of Conduct, no informed consent is needed for this study. This procedure, including the need for informed consent, was waived and approved by the Medical Ethics Committee of the Erasmus MC (approval number MEC 02.953).

## Results

### Patient and tumor characteristics

In total 394 patients were included in this breast cancer cohort and demographics and tumor characteristics are shown in Table 1. Most patients (n = 297; 75%) were between 41 and 70 years of age. At diagnosis the majority had no lymph node metastasis (n = 233; 59%) and a T1 tumor (n = 267, 68%). In the entire cohort 85% was ER-positive, 74% PR-positive, 12% had a HER2 amplification and 11% had neither a HER2 amplification nor hormone receptor expression (TNBC). Adjuvant chemotherapy was administered in 158 patients (40%) while 179 (45%) received adjuvant hormone therapy.

**Table 1:** Baseline characteristics and the association with mGluR1 expression in the entire cohort.

	No. of patients		mGluR1 expression				*p* value^§^
			Positive		Negative		
	n	(%)	n	(%)	n	(%)	
**All patients**	394	(100.0)	219	(55.6)	175	(44.4)	
**Age**							0.248^#^
≤ 40	42	(10.7)	23	(54.8)	19	(45.2)	
> 40− ≤ 55	174	(44.2)	91	(52.3)	83	(47.7)	
> 55− ≤ 70	123	(31.2)	71	(57.7)	52	(42.3)	
> 70	55	(14.0)	34	(61.8)	21	(38.2)	
**T-stage**							0.902^#^
T1	267	(67.8)	147	(55.1)	120	(44.9)	
T2	111	(28.2)	64	(57.7)	47	(42.3)	
T3	11	(2.8)	5	(45.5)	6	(54.5)	
T4	5	(1.3)	3	(60.0)	2	(40.0)	
**N-stage**							0.156^#^
N0	233	(59.1)	126	(54.1)	107	(45.9)	
N1	113	(28.7)	60	(53.1)	53	(46.9)	
N2	48	(12.2)	33	(68.8)	15	(31.3)	
**Tumor grade**							< 0.001^#^
1	114	(28.9)	91	(79.8)	23	(20.2)	
2	192	(48.7)	92	(47.9)	100	(52.1)	
3	88	(22.3)	36	(40.9)	52	(59.1)	
ER status*							< 0.001
pos	336	(85.3)	201	(59.8)	135	(40.2)	
neg	58	(14.7)	18	(31.0)	40	(69.0)	
PR status*							< 0.001
pos	291	(73.9)	178	(61.2)	113	(38.8)	
neg	103	(26.1)	41	(39.8)	62	(60.2)	
HER2 status*							0.969
pos	47	(11.9)	26	(55.3)	21	(44.7)	
neg	347	(88.1)	193	(55.6)	154	(44.4)	
**TN status**							< 0.001
yes	44	(11.2)	11	(25.0)	33	(75.0)	
no	350	(88.8)	208	(59.4)	142	(40.6)	
**Chemotherapy**							0.014
Yes	158	(40.1)	76	(48.1)	82	(51.9)	
No	236	(59.9)	143	(60.6)	93	(39.4)	
**Hormonal therapy**							0.126
Yes	179	(45.4)	107	(59.8)	72	(40.2)	
No	215	(54.6)	112	(52.1)	103	(47.9)	

*As retrieved from TMA.

^**§**^*p* value for chi-square test.

^#^chi-square trend test performed.

### mGluR1 is frequently expressed in breast cancer tissue

mGluR1 membrane staining was positive in more than half (n = 219; 56%) of the breast cancer tissues (Table 1). There was a diffuse membrane staining in 37% of the mGluR1 positive tumors and the remaining 63% had an apical staining pattern. In most mGluR1 positive tumors (85%) the quantity of mGluR1 stained tumor cells was ≥ 50% while in 62% of these tumors the quantity of the mGluR1 expressing tumor cells was ≥ 80%. Examples of mGluR1 protein staining are shown in Fig. 2. Testing for association of mGluR1 expression with clinicopathological characteristics showed that mGluR1 expression was associated with a lower tumor grade and with ER-positive and PR-positive tumors (Table 1). There was no relationship with age, nodal involvement and tumor size. The percentage of tumor cells expressing mGluR1 differed between breast cancer subgroups. ER-positive and PR-positive tumors expressed mGluR1 (~ 60%) almost twice as frequent as ER or PR negative tumors (31% and 40% respectively). Among TNBC a quarter expressed mGluR1 contrary to 59% in non-TNBC tumors. No difference was observed in mGluR1 expression in relation to HER2 status.

Invasive ductal carcinoma was the most prevalent histological subtype (77%) and expressed mGluR1 in 54% while the less frequent tubular (4.6%) and papillary (2.0%) breast cancer subtypes were almost exclusively mGluR1 positive. All tumors of the medullary subtype (1.3%) were negative for mGluR1 (Additional file 2).

### No association of mGluR1 expression with prognosis in the entire cohort

All 394 patients were included for survival analysis. The median follow-up time was 94 months (range 5–181 months). All known prognostic factors and mGluR1 expression were included in the analysis of MFS (Table 2). Univariate analysis showed that expression of mGluR1 protein was not associated with MFS (HR 1.65, 95% CI 0.89–3.04, *p* = 0.111) in the entire cohort. To evaluate which of the clinical parameters are independently associated with MFS, we used multivariate Cox regression analysis and this showed that a larger tumor size, more involvement of lymph nodes and no-treatment with chemotherapy were prognostic factors for worse outcome. Also, mGluR1 expression was not associated with OS in univariate analysis (HR 1.33, 95% CI 0.98–3.13, *p* = 0.285) (Additional file 3). Additional file 4 shows the relation between mGluR1 expression and MFS and OS, depicted in Kaplan–Meier curves. We did not find a relation between a higher intensity of mGluR1 staining or a higher percentage of stained tumor cells and prognosis in the entire cohort (*p* = 0.057 and *p* = 0.82 respectively).

**Table 2:** Univariate and multivariate analysis of MFS in the entire cohort.

	Univariate analysis			Multivariate analysis		
	HR	95% CI	*p* value	HR	95% CI	*p* value
**Age**						
> 55 versus ≤ 55	0.77	0.42–1.41	0.396	0.41	0.16–1.05	0.062
**T-stage**						
T2-4 versus T1	5.11	2.78–9.42	< 0.001	3.31	1.68–6.51	0.001
**N-stage**						
N1 versus N0	4.19	1.97–8.90	< 0.001	4.04	1.60–10.17	0.003
N2 versus N0	8.83	4.00–19.47	< 0.001	7.47	2.86–19.54	< 0.001
**Tumor grade**						
2 versus 1	1.81	0.76–4.31	0.180	1.23	0.49–3.08	0.665
3 versus 1	4.68	1.99–11-02	< 0.001	2.03	0.75–5.48	0.164
ER status*						
Pos versus neg	0.41	0.22–0.79	0.007	0.26	0.04–1.59	0.146
PR status*						
Pos versus neg	0.55	0.30–0.99	0.047	0.53	0.18–1.56	0.249
HER2 status*						
Pos versus neg	2.07	1.00–4.28	0.051	1.37	0.48–3.91	0.558
**TN status**						
Yes versus no	1.92	0.93–3.97	0.080	0.60	0.13–2.99	0.530
**mGluR1**						
Pos versus neg	1.65	0.89–3.04	0.111			
**Chemotherapy**						
Yes versus no	1.75	0.98–3.13	0.058	0.22	0.08–0.59	0.003
**Hormonal therapy**						
Yes versus no	1.88	1.04–3.38	0.036	1.86	0.58–5.97	0.297

*As retrieved from TMA.

mGluR1 was not added in the multivariate base model with known prognostic markers because no significance was achieved in univariate regression analysis.

### Unfavorable outcome with mGluR1 expression in different breast cancer subgroups

We subsequently examined the prognostic value of mGluR1 expression in different breast cancer subgroups. mGluR1 expression was associated with a significantly shorter MFS in ER-negative breast cancer and in TNBC (Table 3).

**Table 3:** Prognostic value of mGluR1 expression in clinically relevant breast cancer subgroups.

Subgroups	n	mGluR1 Positive versus Negative		
		HR	95% CI	*p* value
ER positive	336	1.23	0.60–2.52	0.577
ER negative	58	7.09	2.17–23.15	0.001
HER2 positive	47	7.26	0.91–58.12	0.062
HER2 negative	347	1.31	0.68–2.53	0.427
TN no	350	1.51	0.75–3.05	0.251
TN yes	44	5.23	1.40–19.60	0.014

Univariate cox-regression analysis of MFS in different breast cancer subgroups (as retrieved from TMA).

**Figure 3:**
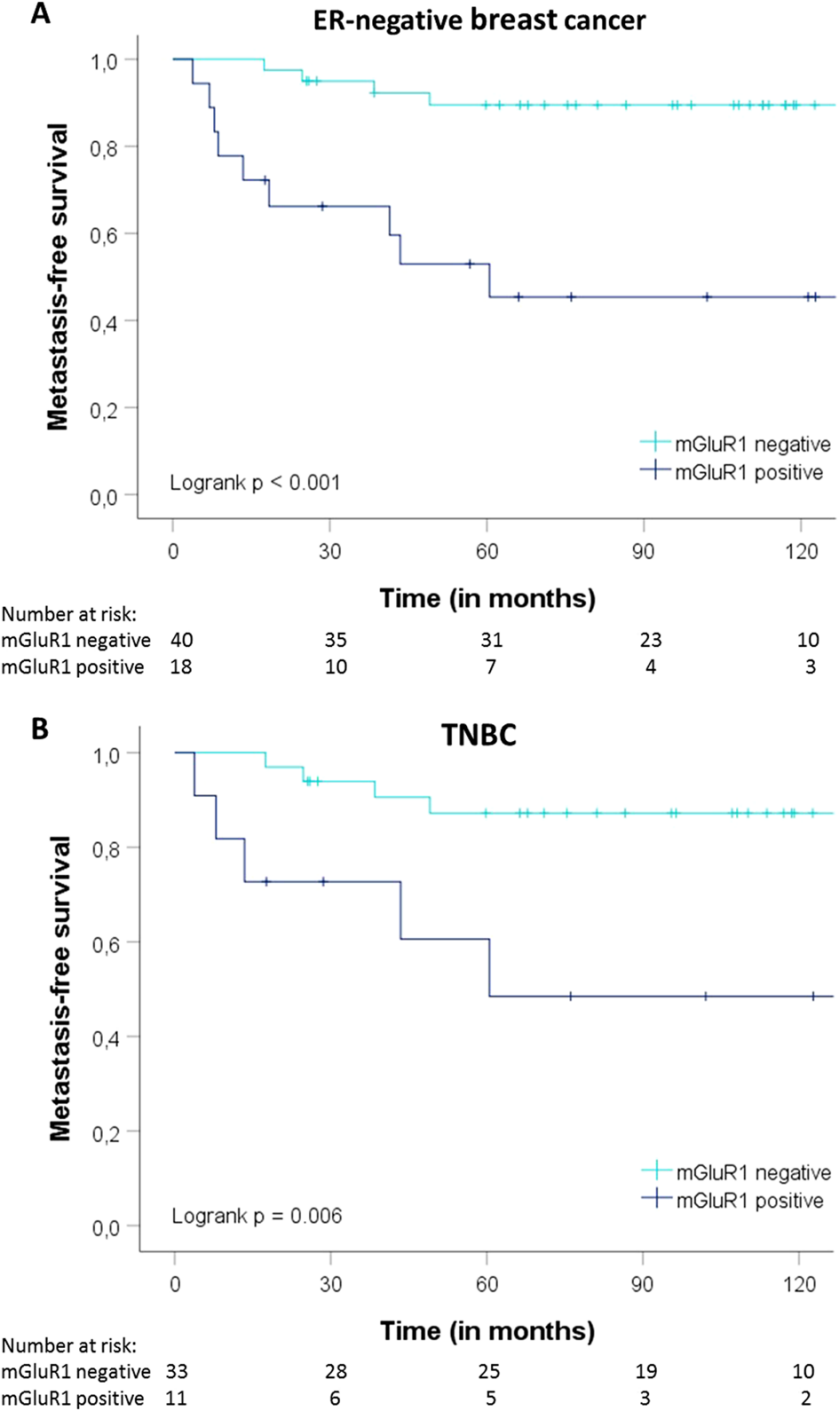
Kaplan–Meier estimates of MFS as a function of mGluR1 expression in ER-negative breast cancer and TNBC. Kaplan–Meier estimates of MFS: (**a**) ER-negative breast cancer; and (**b**) TNBC. Patients were divided into two groups based on mGluR1 expression. Positive mGluR1 expression is depicted in dark blue lines and negative mGluR1 expression is depicted in light blue lines.

Eighteen of the 58 ER-negative tumors (31%) were mGluR1-positive. In ER-negative tumors, mGluR1 expression was associated with higher age and more lymph node involvement (Additional file 5). Most patients (n = 43) in this ER-negative subgroup were treated with adjuvant chemotherapy. In univariate Cox regression analysis, mGluR1 expression and involvement of more lymph nodes were significantly associated with a shorter MFS (Table 4a). In the multivariate model, when corrected for known prognostic factors, only mGluR1 expression remained as an independent predictor for a poor MFS (HR 5.00, 95% CI 1.03–24.35, *p* = 0.046). Figure 3a shows Kaplan–Meier estimates of MFS. Poor OS was significantly associated with involvement of more lymph nodes and mGluR1 expression in univariate analysis (HR 7.46, 95% CI 1.91–29.16, *p* = 0.004). However, none of the predictors were significant in a multivariate model (mGluR1 HR 5.24, 95% CI 0.90–30.60, *p* = 0.066) (Additional file 6a; for Kaplan–Meier estimates of OS see Additional file 7a).

**Table 4:** Univariate and multivariate analysis of MFS in ER-negative breast cancer and TNBC.

**(A)**						
ER-negative breast cancer	Univariate analysis			Multivariate analysis		
	HR	95% CI	*p* value	HR	95% CI	*p* value
**Age**						
> 55 versus ≤ 55	1.51	0.51–4.49	0.459	0.76	0.13–4.57	0.765
**T-stage**						
T2-3 versus T1	1.83	0.61–5.45	0.279	1.63	0.37–7.12	0.517
**N-stage**						
N1-N2 versus N0	5.26	1.62–17.13	0.006	4.03	0.93–17.47	0.063
**Tumor grade**						
3 versus 1–2	1.46	0.40–5.31	0.568	1.68	0.42–6.69	0.462
HER2 status*						
Pos versus neg	1.69	0.52–5.48	0.385	1.49	0.42–5.31	0.543
**TN status**						
Yes versus no	0.67	0.21–2.17	0.504			
**mGluR1**						
Pos versus neg	7.09	2.17–23.15	0.001	5.00	1.03–24.35	0.046
**Chemotherapy**						
Yes versus no	0.357	0.10–1.06	0.065	0.43	0.08–2.26	0.317

**(B)**						
TNBC	Univariate analysis			Multivariate analysis		
	HR	95% CI	*p* value	HR	95% CI	*p* value
**Age**						
> 55 versus ≤ 55	1.16	0.31–4.32	0.826	1.24	0.07–21.10	0.881
**T-stage**						
T2-3 versus T1	2.80	0.70–11.25	0.146	3.13	0.38–25.76	0.288
**N-stage**						
N1-N2 versus N0	5.02	1.25–20.14	0.023	2.44	0.24–24.88	0.451
**Tumor grade**						
3 versus 1–2	0.71	0.18–2.85	0.631	0.64	0.14–2.97	0.571
**mGluR1**						
Pos versus neg	5.23	1.40–19.60	0.014	8.60	1.06–70.19	0.044
**Chemotherapy**						
Yes versus no	0.67	0.17–2.67	0.566	1.67	0.14–20.39	0.688

*As retrieved from TMA.

Univariate and multivariate analysis of MFS in: **a** ER-negative breast cancer. TN status was excluded in multivariate cox-regression analysis due to correlation with HER2 status; **b** TNBC.

Forty-four patients had TNBC with mGluR1 expression in 11 patients (25%) (Additional file 8). Age and adjuvant chemotherapy associated significantly with mGluR1 expression. In univariate analysis mGluR1 expression and involvement of more lymph nodes were associated with a shorter MFS, while in the multivariate model only mGluR1 expression remained significant (HR 8.60, 95% CI 1.06–20.39, *p* = 0.044) (Table 4b). Kaplan–Meier estimates of MFS are shown in Fig. 3b. Only mGluR1 expression was significantly associated with OS in both univariate (HR 8.61, 95% CI 1.55–47.81, *p* = 0.014) and multivariate analysis (HR 16.07, 95% CI 1.16–223.10, *p* = 0.039) (Additional file 6b; for Kaplan–Meier estimates of OS see Additional file 7b).

In PR-negative breast cancer, mGluR1 expression was associated with unfavorable MFS in both univariate and multivariate analysis (additional file 9). As shown in Table 3, there was no prognostic value of mGluR1 expression in ER-positive breast cancer. Additional file 10 shows Kaplan–Meier estimates of MFS and OS as a function of mGluR1 expression in ER-positive breast cancer.

### GRM1 RNA expression in TNBC is not associated with outcome

We also performed survival analysis for GRM1 RNA expression in TNBC patients from several cohorts. First, we studied Affymetrix U133-A array data in a cohort of 866 breast cancer patients, including 142 with TNBC^20^. In the entire cohort, the three GRM1 probes were low responsive with barely any variation in gene expression, questioning the validity of the probes. Therefore, we did not use this cohort. Then we studied RNAseq data in TNBC patients from three different breast cancer cohorts. No significant relationship between GRM1 RNA expression level and outcome in TNBC was observed^21–23^ (additional file 11).

## Discussion

In the current study we demonstrate that mGluR1 expression is an independent, unfavorable prognostic factor in ER-negative breast cancer and in TNBC. To the best of our knowledge, this is the first study that shows an association between outcome and mGluR1 protein expression in hormone receptor negative breast cancer and TNBC.

This primary breast cancer cohort is a representative cohort, containing 15% ER-negative tumors and 11% TNBC^24^. Overall, we found that mGluR1 is frequently expressed in breast cancer with mGluR1 positivity in 56% of the tumors and this is in line with previous studies in which a similar percentage positivity was reported^8,26^. Also, a consistent distribution is seen regarding the histological subtypes, in which half of the invasive ductal carcinomas express mGluR1 and one third of the invasive lobular carcinomas^26^. Expression of mGluR1 in relation to hormone receptor status, with ER/PR-positive tumors having double the amount of mGluR1 expression than hormone receptor negative tumors, was also concordant to a previous study^8^. This last observation suggests that expression of mGluR1 may be influenced by hormonal stimulation. Alternatively, the difference in mGluR1 expression may merely reflect cell type specific differences between hormone negative and positive tumors.

Several lines of evidence suggest that mGluR1 contributes to malignant behavior in breast cancer. First, mGluR1 overexpression transforms cells to a malignant phenotype with increased proliferation and invasion^13,26,27^. Second, reduction of mGluR1 activity in TNBC results in tumor suppression and reduced angiogenesis^18^. Finally, mGluR1 can operate as a regulator of inflammation by creating an immunosuppressive tumor microenvironment resulting in inhibition of antitumor activity^28,29^. With regard to the clinical connection between expression of mGluR1 and aggressiveness, in the entire cohort there was no association between the expression of mGluR1 and MFS or OS. However, in ER-negative breast cancer, and the aggressive TNBC subset, mGluR1 expression marks cancer with unfavorable outcome. Based on RNA (GRM1) expression, Mehta et al. reported that low GRM1 expression was associated with longer MFS in tamoxifen treated ER-positive breast cancer^8^. Although these authors also observed a negative correlation between mGluR1 expression and prognosis, our data do not confirm an association between mGluR1 expression and prognosis in ER-positive breast cancer. Also in ER-positive patients receiving adjuvant tamoxifen we did not find such correlation (*p* = 0.29). This difference may be explained by methodological issues as Mehta et al.^8^ studied GRM1 at the RNA level whereas in our study mGluR1 was determined at the protein level.

To support our findings, we performed survival analysis for GRM1 RNA expression in TNBC patients from several cohorts but no significant relationship with outcome was observed^20–23^. However, datasets are relatively small and differences in protein expression are not always correlated to RNA expression levels. More studies are needed to clarify the relation between mGluR1 expression, at both the mRNA and protein level, and prognosis in breast cancer. In addition, the major factors that regulate mGluR1 protein expression need further study.

Why mGluR1 expression is linked to poor outcome in hormone receptor negative tumors only, is not understood. Differences in downstream activation of mGluR1 through glutamate between hormone sensitive versus hormone refractory tumors may explain the difference in prognostic value of mGluR1 in hormone receptor positive versus the important hormone receptor negative breast cancer subgroup^30–32^. However, differences in immune suppression may be an alternative explanation. TNBC is a heterogeneous group of cancers^33^, and it is also conceivable that mGluR1 is a marker of a more aggressive subtype within TNBC.

New therapies are an urgent medical need, especially for the more aggressive TNBC that can only be treated with cytotoxic chemotherapy. However, also for other breast cancer subgroups new targets are needed as a significant number of tumors will develop resistance to current therapies^34,35^. Several studies have shown that mGluR1 is a potential therapeutic target in breast cancer^13,26^. Most of these studies have been performed in TNBC only. A clinically available drug named riluzole is a glutamate release inhibitor and is currently under investigation as a potential treatment for breast cancer. However, in breast cancer cells the effect of riluzole is not dependent on mGluR1 and recently a riluzole trial in advanced melanoma patients failed to demonstrate an improved outcome^29,36,37^. Thus, directly targeting mGluR1 rather than its ligand glutamate may be a better approach, as has been demonstrated by using a specific mGluR1 inhibitor in mice orthotopically carrying a TNBC xenograft^13^. Another approach may be the use of mGluR1 targeting antibodies that either effectively block the receptor, elicit an immune response and/or deliver a toxic payload to mGluR1 expressing breast cancer cells.

Our study has several limitations. First, this is an older cohort, starting in 2000. The advantage of an older cohort is the maturity of the follow-up data. However, over time treatment strategies have changed, affecting outcome in breast cancer patients diagnosed today. Another limitation is the relatively small sample size of some breast cancer subgroups, in particular TNBC, for which our conclusion should be validated in a larger independent cohort.

## Conclusions

Our study shows that mGluR1 expression is highly prevalent in most breast cancer subgroups. In addition, we show that in patients with ER-negative breast cancer or TNBC mGluR1 is an unfavorable prognostic marker independent of known clinical and histopathological prognostic factors. Clearly, independent validation of this prognostic value is required. Nonetheless, our data imply that mGluR1 has the potential to be a relevant target for the treatment of breast cancer.

## Supplementary Information

Supplementary Information.

## Data Availability

All data and materials are available and can be obtained from the corresponding author upon reasonable request.

## Abbreviations

ALS: Amyotrophic lateral sclerosis
ATA: Automated Tissue Arrayer
CHO: Chinese hamster ovary
CI: Confidence interval
ER: Estrogen receptor
FFPE: Formalin-fixed paraffin-embedded
FISH: Fluorescence in situ hybridization
GRM1: Metabotropic glutamate receptor 1 (gene)
HER2: Human epidermal growth factor type 2 receptor
HR: Hazard ratio
IDC: Invasive ductal carcinoma
IHC: Immunohistochemistry
ILC: Invasive lobular carcinoma.
MAPK: Mitogen-activated protein kinase
MFS: Metastasis-free survival
mGluR1: Metabotropic glutamate receptor 1 (protein)
OS: Overall survival
PR: Progesterone receptor
TMA: Tissue microarray
TNBC: Triple-negative breast cancer
