# Supplementary material for: Metabotropic glutamate receptor 1 is associated with unfavorable prognosis in ER-negative and triple-negative breast cancer
Source: Sci Rep. 2020 Dec 18;10:22292. doi: 10.1038/s41598-020-79248-4 (PMC7749122; doi:10.1038/s41598-020-79248-4)
Supplement: Supplementary file 1 — Supplementary Information. [file 41598_2020_79248_MOESM1_ESM.pdf]

Metabotropic glutamate receptor 1 is associated with unfavorable prognosis in ER-negative and triple-negative breast cancer

Anna E.M. Bastiaansen, A. Mieke Timmermans, Marcel Smid, Carolien H.M. van Deurzen, Esther S.P. Hulsenboom, Wendy J.C. Prager-van der Smissen, Renée Foekens, Anita M.A.C. Trapman-Jansen, Peter A.E. Sillevis Smitt, Theo M. Luider, John W.M. Martens*, Martijn M. vanDuijn*

*Shared last and corresponding authors

Additional file 1. Primary antibodies used in immunohistochemistry.

Antigen	Antibody	Used concentration	pH antigen retrieval	company
ER	1D5	1:50	9	Agilent Dako
PR	PgR 636	1:100	9	Agilent Dako
mGluR1	ab27192	1:8	6	Abcam
HER2	Herceptest	Directly from kit	See kit	Agilent Dako

Additional file 2. Histological subtypes and mGluR1 expression.

	No. of patients		mGluR1 expression				p-value
			Positive (n=219)		Negative (n=175)		
	n	(%)	n	(%)	n	(%)	<0.001
**Histological subtype (n=394)**	394	(100.0)	219	(55.6)	175	(44.4)	
**IDC**	305	(77.4)	164	(53.8)	141	(46.2)	
**ILC**	27	(6.9)	10	(37.0)	17	(63.0)	
**IDC/ILC**	16	(4.1)	10	(62.5)	6	(37.5)	
**Medullary**	5	(1.3)	0	(0.0)	5	(100.0)	
**Mucinous**	5	(1.3)	3	(60.0)	2	(40.0)	
**Tubular**	18	(4.6)	17	(94.4)	1	(5.6)	
**Papillary**	8	(2.0)	8	(100.0)	0	(0.0)	
**Other**	10	(2.5)	7	(70.0)	3	(30.0)	

p-value for comparison within the histological subtypes.

Abbreviations: IDC= Invasive ductal carcinoma, ILC= Invasive lobular carcinoma.

Additional file 3. Univariate and multivariate analysis of OS in the entire cohort.

		Univariate analysis			Multivariate analysis		
		**HR**	**95% CI**	**p-value**	**HR**	**95% CI**	**p-value**
**Age**	>55 vs ≤55	1.81	1.09-3.00	0.022	1.00	0.49-2.04	0.995
**T-stage**	T2-4 vs T1	2.97	1.79-4.93	<0.001	1.95	1.11-3.43	0.021
**N-stage**	N1 vs N0	2.07	1.13-3.79	0.018	2.12	0.96-4.70	0.063
	N2 vs N0	4.58	2.46-8.52	<0.001	3.47	1.53-7.89	0.003
**Tumor grade**	2 vs 1	1.87	0.91-3.84	0.088	1.42	0.67-3.01	0.354
	3 vs 1	3.58	1.69-7.57	0.001	3.08	1.29-7.38	0.012
**ER status***	Pos vs neg	0.79	0.40-1.55	0.488	0.40	0.08-2.00	0.266
**PR status***	Pos vs neg	0.71	0.41-1.24	0.232	0.52	0.23-1.17	0.113
**HER2 status***	Pos vs neg	1.59	0.78-3.24	0.201	1.00	0.37-2.71	0.995
**TN status**	Yes vs no	0.91	0.39-2.10	0.816	0.33	0.06-1.66	0.117
**mGluR1**	Pos vs neg	1.33	0.79-2.24	0.285			
**Chemotherapy**	Yes vs no	0.80	0.48-1.34	0.398	0.27	0.13-0.59	0.001
**Hormonal therapy**	Yes vs no	2.10	1.25-3.55	0.005	1.79	0.75-4.28	0.188

* As retrieved from TMA.

mGluR1 was not added in the multivariate base model with known prognostic markers because no significance was achieved in univariate regression analysis .

Additional file 4. Kaplan-Meier estimates of MFS and OS as a function of mGluR1 expression in the total cohort.

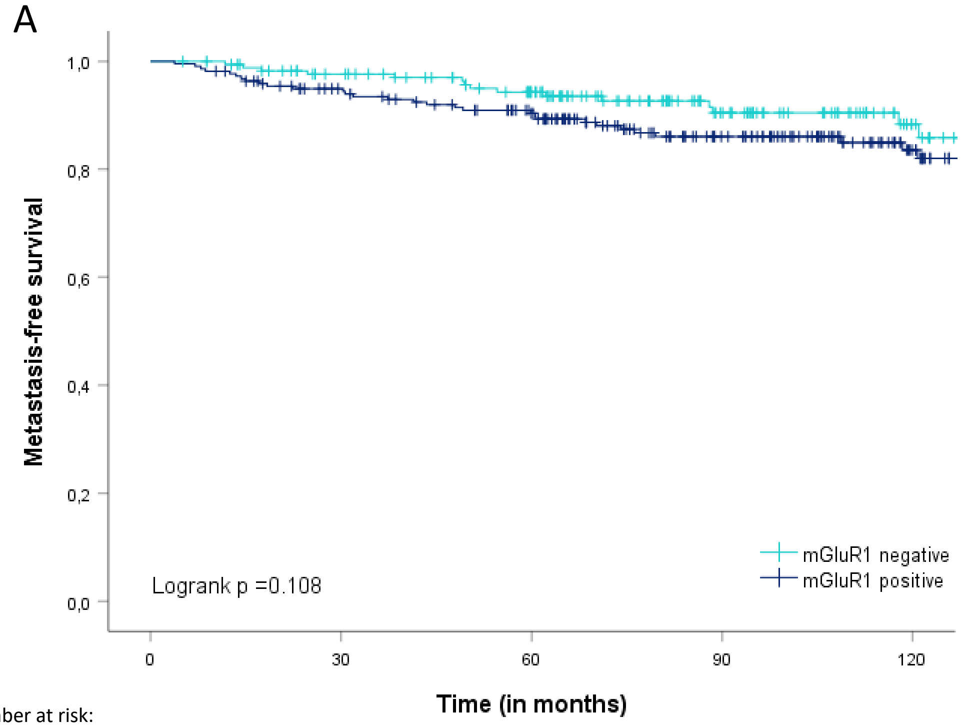

Number at risk:					
mGluR1 negative	175	157	129	79	36
mGluR1 positive	219	193	167	110	56

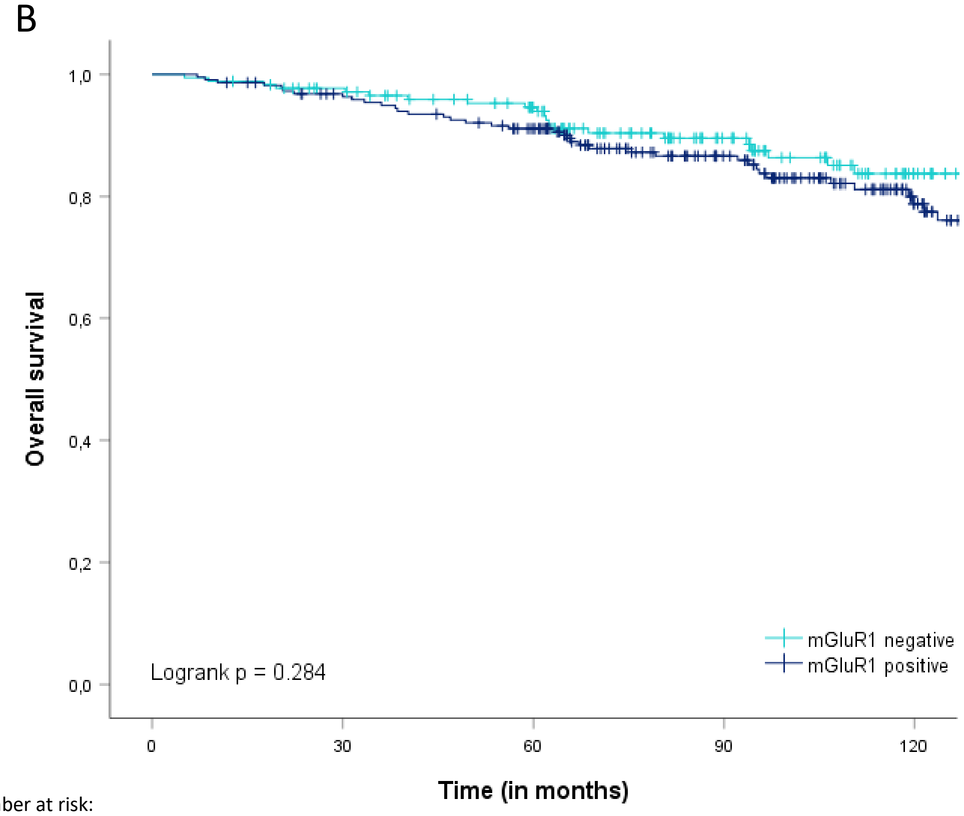

Number at risk:					
mGluR1 negative	175	163	139	92	46
mGluR1 positive	219	204	183	125	64

Patients were divided into two groups based on mGluR1 expression. Positive mGluR1 expression is depicted in dark blue lines and negative mGluR1 expression is depicted in light blue lines. **a** Kaplan-Meier Curve of MFS. **b** Kaplan-Meier Curve of OS.

Additional file 5. ER-negative breast cancer characteristics and the association with mGluR1 expression.

	No. of patients		mGluR1 expression				p-value[§]
			Positive		Negative		
	n	(%)	n	(%)	n	(%)	
**All patients**	58	(100)	18	(31)	40	(69)	
**Age**							0.015
≤55	36	(62.1)	7	(19.4)	29	(80.6)	
>55	22	(37.9)	11	(50.0)	11	(50.0)	
**T-stage**							0.664
T1	33	(56.9)	22	(66.7)	22	(66.7)	
T2-T3	25	(43.1)	18	(72.0)	18	(72.0)	
**N-stage**							0.040
N0	37	(63.8)	8	(21.6)	29	(78.4)	
N1-N2	21	(36.2)	10	(47.6)	11	(52.4)	
**Tumor grade**							0.983
1-2	16	(27.6)	5	(31.3)	11	(68.8)	
3	42	(72.4)	13	(31.0)	29	(69.0)	
**HER2 status***							0.181
Pos	13	(22.4)	6	(46.2)	7	(53.8)	
Neg	45	(77.6)	12	(26.7)	33	(73.3)	
**TN status**							0.078
Yes	44	(75.9)	11	(25.0)	33	(75.0)	
No	14	(24.1)	7	(50.0)	7	(50.0)	
**Chemotherapy**							0.001
Yes	43	(74.1)	8	(18.6)	35	(81.4)	
No	15	(25.9)	10	(66.7)	5	(33.3)	

* As retrieved from TMA. [§] p-value for chi-square test.

No T-stage 4 present in this subgroup. Tumor grade 1 and 2 were combined due to small number of cases. PR-status and hormonal therapy were excluded for analysis due to limited number of cases.

Additional file 6. Univariate and multivariate analysis of OS in ER-negative breast cancer and TNBC.

A

**ER-negative breast cancer**		**Univariate analysis**			**Multivariate analysis**		
		**HR**	**95% CI**	**p-value**	**HR**	**95% CI**	**p-value**
**age**	>55 vs ≤55	1.87	0.54-6.47	0.324	2.01	0.25-16.32	0.513
**T-stage**	T2-3 vs T1	1.53	0.44-5.28	0.506	1.06	0.21-5.50	0.944
**N-stage**	N1-N2 vs N0	5.23	1.35-20.29	0.017	4.98	0.87-28.57	0.072
**Tumor grade**	3 vs 1-2	3.75	0.48-29.64	0.210	4.22	0.49-36.35	0.190
**HER2 status***	Pos vs neg	2.43	0.69-8.63	0.169	1.85	0.48-7.08	0.371
**TN status**	Yes vs no	0.46	0.13-1.64	0.233			
**mGluR1**	Pos vs neg	7.46	1.91-29.16	0.004	4.76	0.83-27.13	0.079
**Chemotherapy**	Yes vs no	0.48	0.14-1.69	0.252	1.27	0.19-8.31	0.803

B

**TNBC**		**Univariate analysis**			**Multivariate analysis**		
		**HR**	**95% CI**	**p-value**	**HR**	**95% CI**	**p-value**
**age**	>55 vs ≤55	1.52	0.31-7.53	0.610	1.46	0.06-36.94	0.820
**T-stage**	T2-3 vs T1	2.62	0.48-14.39	0.267	4.38	0.33-57.87	0.262
**N-stage**	N1-N2 vs N0	4.87	0.89-26.76	0.069	1.95	0.11-35.74	0.653
**Tumor grade**	3 vs 1-2	1.65	0.19-14.12	0.648	1.19	0.12-12.14	0.884
**mGluR1**	Pos vs neg	8.61	1.55-47.81	0.014	16.07	1.16-223.10	0.039
**Chemotherapy**	Yes vs no	0.64	0.12-3.48	0.602	2.49	0.13-48.87	0.549

* As retrieved from TMA.

Univariate and multivariate analysis of OS in: **a** ER-negative breast cancer. TN was excluded in multivariate cox-regression analysis due to correlation with HER2 status; **b** TNBC.

Additional file 7. Kaplan-Meier estimates of OS as a function of mGluR1 expression in ER-negative breast cancer and TNBC.

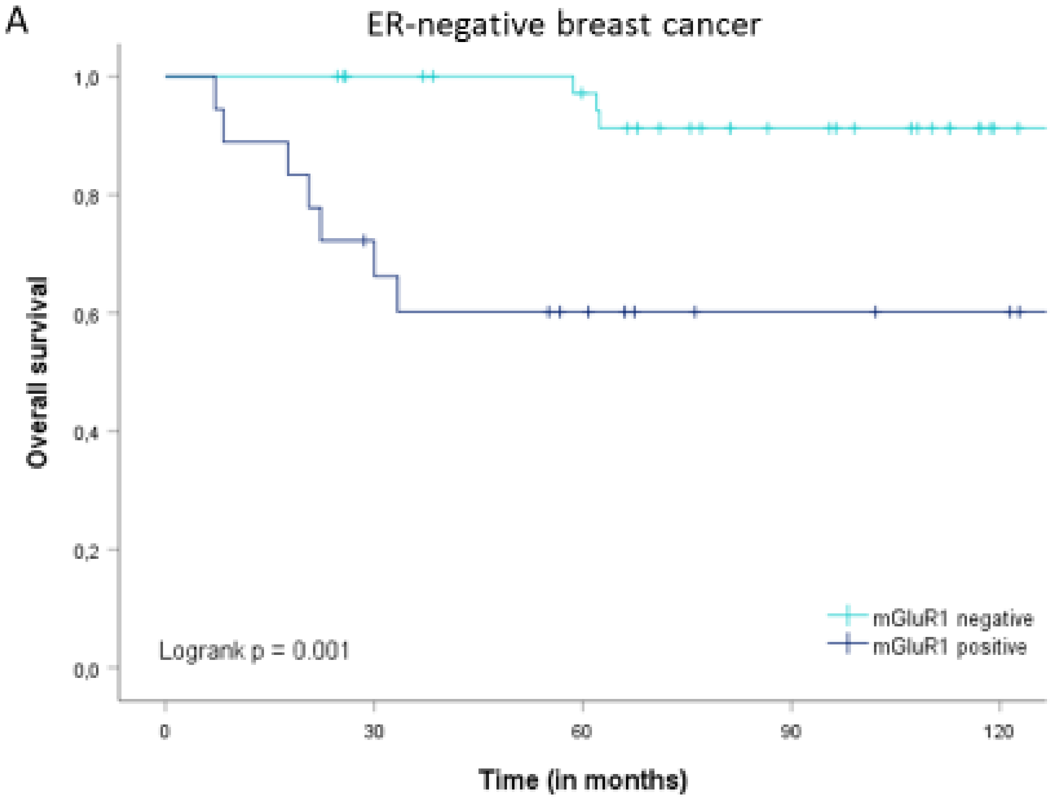

Number at risk:					
mGluR1 negative	40	37	33	24	12
mGluR1 positive	18	12	8	4	3

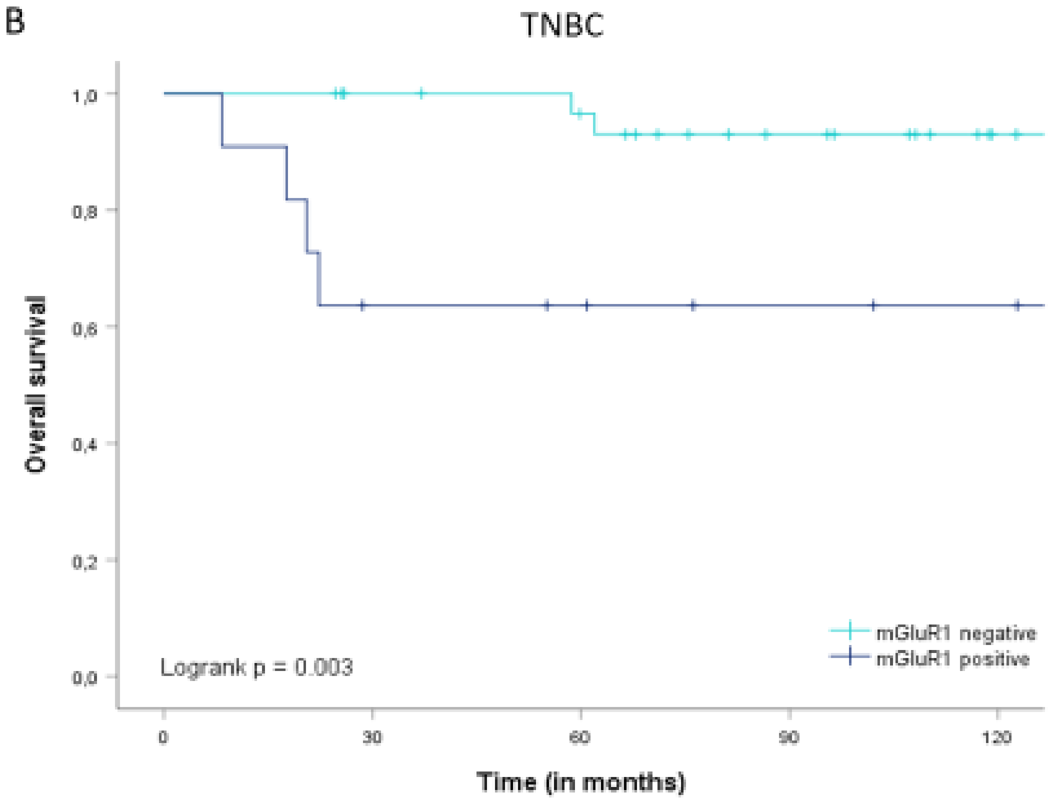

Number at risk:					
mGluR1 negative	33	30	27	20	12
mGluR1 positive	11	6	5	3	2

Kaplan-Meier estimates of OS in: **a** ER-negative breast cancer; and **b** TNBC. Patients were divided into two groups based on mGluR1 expression. Positive mGluR1 expression is depicted in dark blue lines and negative mGluR1 expression is depicted in light blue lines.

Additional file 8. TNBC characteristics and the association with mGluR1 expression.

	No. of patients		mGluR1 expression Positive		Negative		p-value[§]
	n	(%)	n	(%)	n	(%)	
**All patients**	44	(100)	11	(25.0)	33	(75.0)	
**Age**							0.031[‡]
≤55	26	(59.1)	3	(11.5)	23	(88.5)	
>55	18	(40.9)	8	(44.4)	10	(55.6)	
**T-stage**							0.862
T1	23	(52.3)	6	(26.1)	17	(73.9)	
T2-T3	21	(47.7)	5	(23.8)	16	(76.2)	
**N-stage**							0.098
N0	29	(65.9)	5	(17.2)	24	(82.8)	
N1-N2	15	(34.1)	6	(40.0)	9	(60.0)	
**Tumor grade**							1.000[‡]
1-2	11	(25.0)	3	(27.3)	8	(72.7)	
3	33	(75.0)	8	(24.2)	25	(75.8)	
**Chemotherapy**							0.009
Yes	33	(75.0)	5	(15.2)	28	(84.8)	
No	11	(25.0)	6	(54.5)	5	(45.5)	

[§] p-value for chi-square test. [‡] Fisher's exact test

No T-stage 4 present in this subgroup. Tumor grade 1 and 2 were combined due to small number of cases.

Additional file 9. Characteristics and survival analysis in PR-negative breast cancer.

PR-negative breast cancer characteristics and the association with mGluR1 expression.

	**No. of patients**		**mGluR1 expression**				**p-value§**
			**Positive**		**Negative**		
	n	(%)	n	(%)	n	(%)	
**All patients**	103	(100.0)	41	(39.8)	62	(60.2)	
**Age**							0.005
≤55	45	(43.7)	11	(24.4)	34	(75.6)	
>55	58	(56.3)	30	(51.7)	28	(48.3)	
**T-stage**							0.375
T1	65	(63.1)	28	(43.1)	37	(56.9)	
T2-T3	38	(36.9)	13	(34.2)	25	(65.8)	
**N-stage**							0.709
N0	70	(68.0)	27	(38.6)	43	(61.4)	
N1-N2	33	(32.0)	14	(42.4)	19	(57.6)	
**Tumor grade**							0.112
1-2	58	(56.3)	27	(46.6)	31	(53.4)	
3	45	(43.7)	14	(31.1)	31	(68.9)	
**ER status***							0.021
pos	46	(44.7)	24	(52.2)	22	(47.8)	
neg	57	(55.3)	17	(29.8)	40	(70.2)	
**HER2 status***							0.597
pos	20	(19.4)	9	(45.0)	11	(55.0)	
Neg	83	(80.6)	32	(38.6)	51	(61.4)	
**TN status**							0.078
Yes	44	(42.7)	11	(25.0)	33	(75.0)	
No	59	(57.3)	30	(50.8)	29	(49.2)	
**Chemotherapy**							<0.001
Yes	55	(53.4)	12	(21.8)	43	(78.2)	
No	48	(46.6)	29	(60.4)	19	(39.6)	

* As retrieved from TMA. § p-value for chi-square test.
No T-stage 4 present in this subgroup. Tumor grade 1 and 2 were combined due to small number of cases.

Univariate and multivariate analysis of MFS in PR-negative breast cancer.

**PR-negative subtype**		**Univariate analysis**			**Multivariate analysis**		
		**HR**	**95% CI**	**p-value**	**HR**	**95% CI**	**p-value**
**age**	>55 vs ≤55	1.03	0.40-42.70	0.948	0.82	0.16-4.15	0.814
**T-stage**	T2-3 vs T1	2.33	0.89-6.07	0.083	1.93	0.57-6.53	0.290
**N-stage**	N1-N2 vs N0	4.51	1.66-12.19	0.003	4.17	1.17-14.85	0.028
**Tumor grade**	3 vs 1-2	1.95	0.74-5.12	0.177	1.38	0.39-4.91	0.624
**ER status***	Pos vs neg	0.39	0.13-1.19	0.099	0.35	0.03-3.77	0.384
**HER2 status***	Pos vs neg	2.07	0.72-5.95	0.178	1.74	0.16-19.17	0.649
**TN status**	Yes vs no	1.37	0.53-3.59	0.504	1.04	0.07-14.90	0.980
**mGluR1**	Pos vs neg	4.06	1.43-11.53	0.009	4.13	1.16-14.68	0.029
**Chemotherapy**	Yes vs no	0.71	0.27-1.83	0.475	0.32	0.07-1.54	0.154

* As retrieved from TMA.

Kaplan-Meier estimates of MFS as a function of mGluR1 expression in PR-negative breast cancer

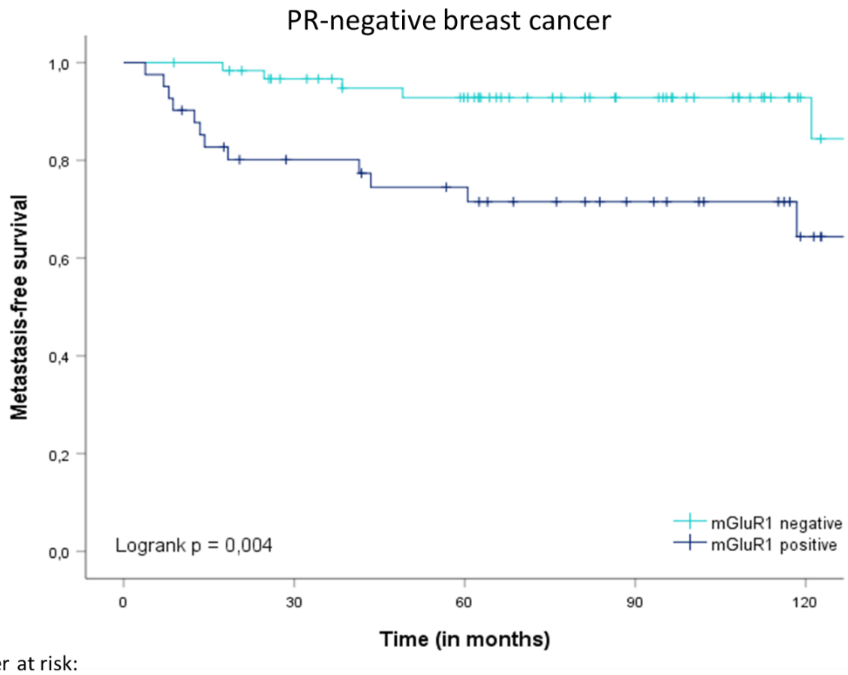

Number at risk:

mGluR1 negative	62	54	46	30	11
mGluR1 positive	41	29	25	17	8

Patients were divided into two groups based on mGluR1 expression. Positive mGluR1 expression is depicted in dark blue lines and negative mGluR1 expression is depicted in light blue lines.

Additional file 10. Kaplan-Meier estimates of MFS and OS as a function of mGluR1 expression in ER-positive breast cancer.

A

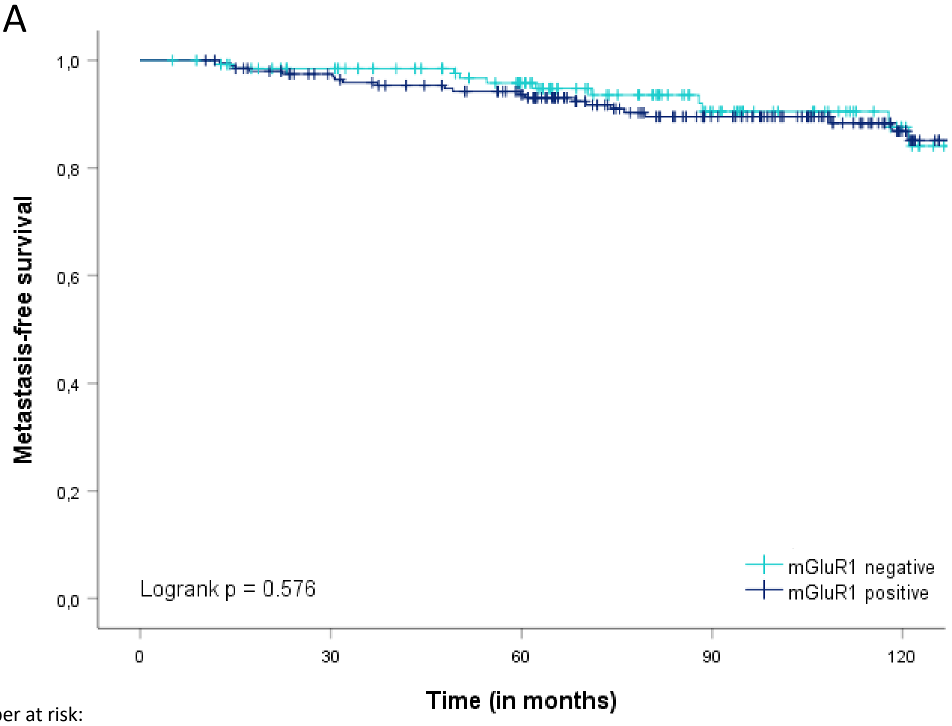

Number at risk:					
mGluR1 negative	135	122	98	56	26
mGluR1 positive	201	183	160	106	53

B

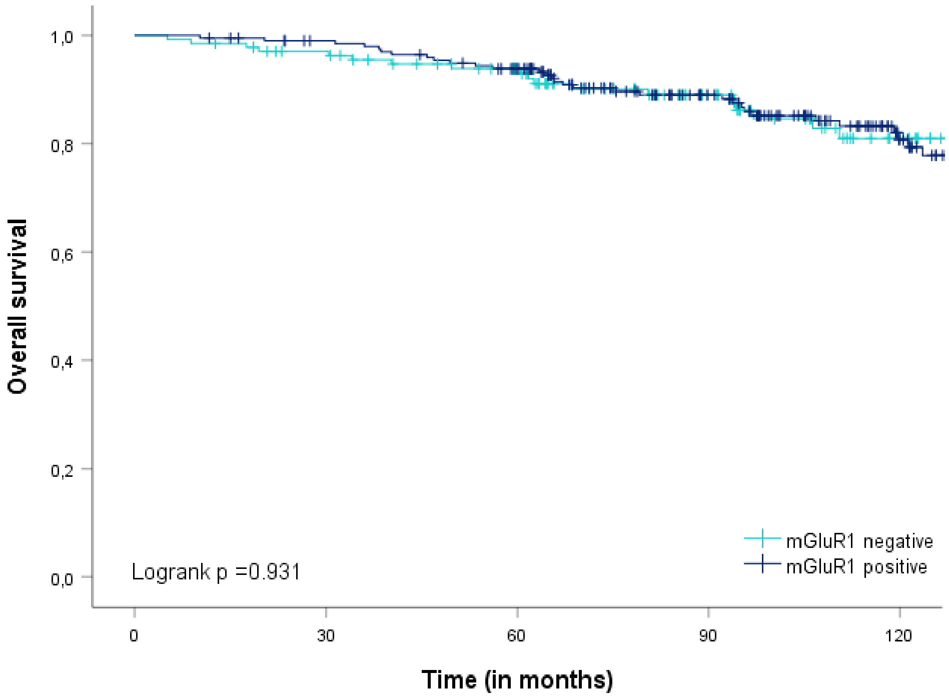

Number at risk:					
mGluR1 negative	135	126	106	68	34
mGluR1 positive	201	192	175	121	61

Patients were divided into two groups based on mGluR1 expression. Positive mGluR1 expression is depicted in dark blue lines and negative mGluR1 expression is depicted in light blue lines. **a** Kaplan-Meier Curve of MFS. **b** Kaplan-Meier Curve of OS.

Additional file 11. Survival analysis for GRM1 gene expression in TNBC patients from several cohorts.

A1

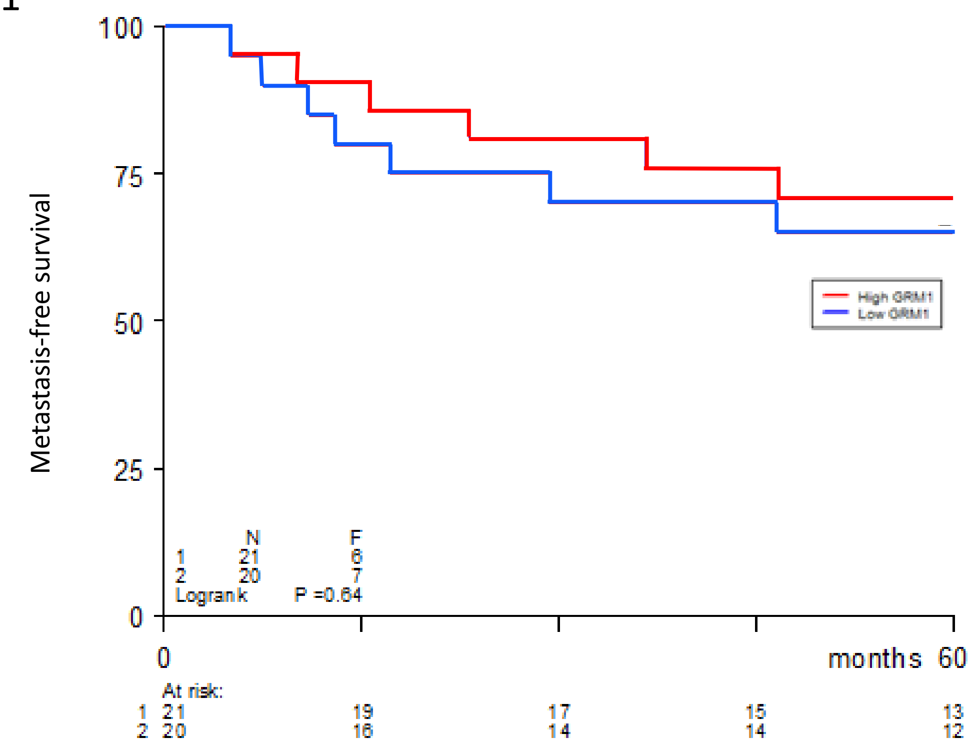

A2

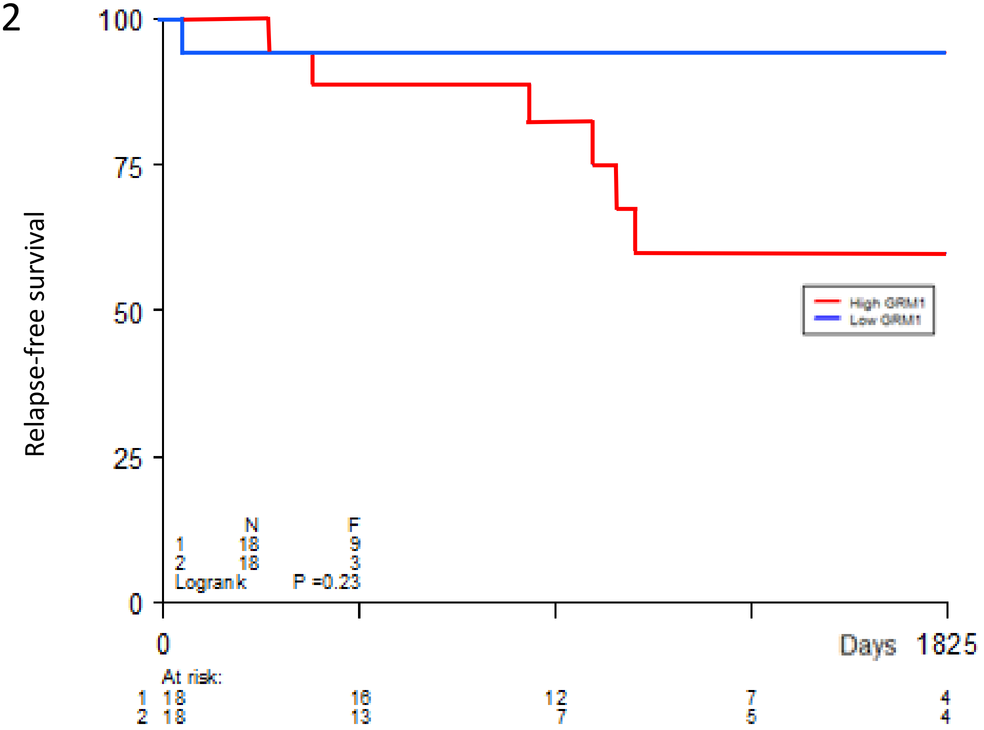

B

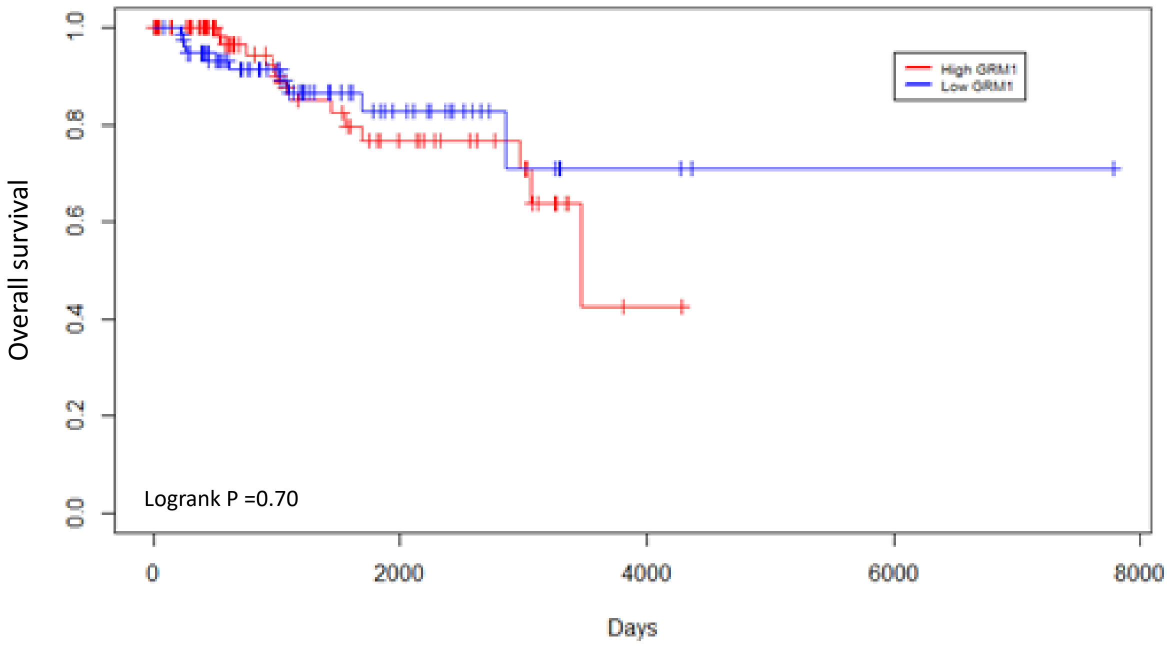

GRM1 RNA expression in different TNBC cohorts. Patients were divided into two groups based on median GRM1 expression. High (above median) GRM1 expression is depicted in red lines and low (below median) GRM1 expression is depicted in blue lines. **a** Survival analysis of RNAseq data in two TNBC cohorts. **a1** Kaplan-Meier Curve of MFS in 41 TNBC patients (Hammerl D. et al. Unpublished data). **a2** Kaplan-Meier Curve of RFS in 36 TNBC patients [23]. **b** Survival analysis in TCGA data of 166 TNBC patients [21,22]. Kaplan-Meier Curve of OS.
